# Identification of markers of sensory quality in ground coffee: an untargeted metabolomics approach

**DOI:** 10.1007/s11306-020-01751-6

**Published:** 2020-12-14

**Authors:** Gabriele Rocchetti, Gian Paolo Braceschi, Luigi Odello, Terenzio Bertuzzi, Marco Trevisan, Luigi Lucini

**Affiliations:** 1 Department for Sustainable Food Process, Università Cattolica del Sacro Cuore, Via Emilia Parmense 84, 29122 Piacenza, Italy; 2 Centro Studi Assaggiatori Società Cooperativa, Galleria V. Veneto, 9, Brescia, Italy; 3 Department of Animal Science, Food and Nutrition, Università Cattolica del Sacro Cuore, Via Emilia Parmense 84, 29122 Piacenza, Italy

**Keywords:** Sensory markers, Food quality, Metabolomics, Food volatiles, UHPLC-QTOF-MS, HS-GC-MS

## Abstract

**Introduction:**

In the last years, consumers increased the demand for high-quality and healthy beverages, including coffee. To date, among the techniques potentially available to determine the overall quality of coffee beverages, metabolomics is emerging as a valuable tool.

**Objective:**

In this study, 47 ground coffee samples were selected during the 2018 Edition of the “International coffee tasting” (ICT) in order to provide discrimination based on both chemical and sensory profiles. In particular, 20 samples received a gold medal (“high quality” group), while lower sensory scores characterized 27 samples (without medal).

**Methods:**

Untargeted metabolomics based on ultra-high pressure liquid chromatography coupled with quadrupole-time-of-flight (UHPLC-QTOF) and head space-gas chromatography coupled with mass spectrometry platforms followed by multivariate statistical approaches (i.e., both supervised and unsupervised) were used to provide new insight into the searching of potential markers of sensory quality.

**Results:**

Several compounds were identified, including polyphenols, alkaloids, diazines, and Maillard reaction products. Also, the headspace/GC-MS highlighted the most important volatile compounds. Polyphenols were scarcely correlated to the sensory parameters, whilst the OPLS-DA models built using typical coffee metabolites and volatile/Maillard compounds possessed prediction values > 0.7. The “high quality” group showed specific metabolomic signatures, thus corroborating the results from the sensory analysis. Overall, methyl pentanoate (ROC value = 0.78), 2-furfurylthiol (ROC value = 0.75), and L-Homoserine (ROC value = 0.74) established the higher number of significant (p < 0.05) correlations with the sensory parameters.

**Conclusion:**

Although *ad-hoc* studies are advisable to further confirm the proposed markers, this study demonstrates the suitability of untargeted metabolomics for evaluating coffee quality and the potential correlations with the sensory attributes.

**Graphic abstract:**

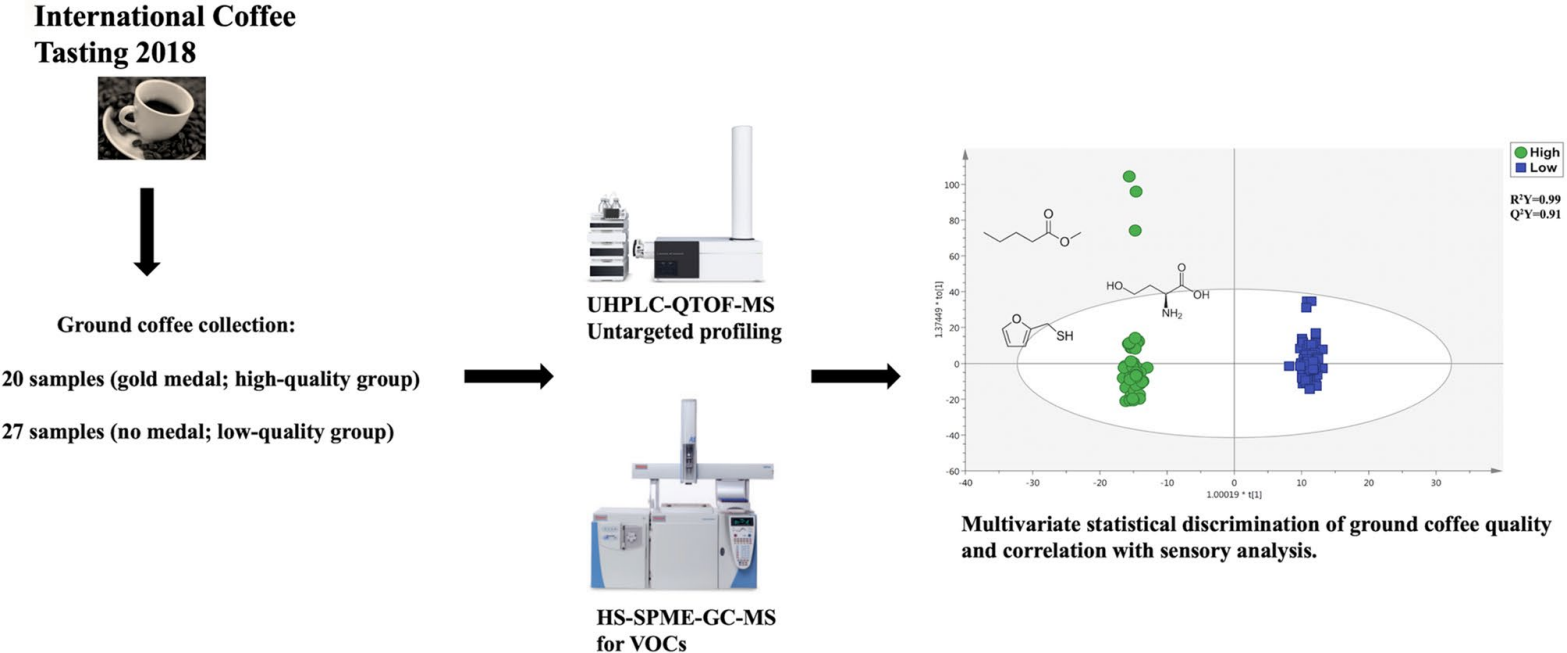

**Supplementary information:**

The online version of this article (10.1007/s11306-020-01751-6) contains supplementary material, which is available to authorized users.

## Introduction

Coffee (*Coffea* sp.) is widely cultivated worldwide, with *Coffea arabica* and *Coffea canephora* variant *robusta* representing the most popular cultivars approximately accounting for 57% and 43% of global coffee production. The sensorial quality of coffee beverage can be affected by several factors, including harvesting and post-harvest, together with roasting and storage conditions. These latter are responsible for the production/degradation of several compounds, including carbohydrates, lipids, and other compounds (Kitzberger et al. 2013, 2014; Cheng et al. 2016; da Rosa et al. 2016). Overall, acidity, body, and sweetness have been described as some of the most important sensorial quality traits for a coffee brew (da Rosa et al. 2016). Also, Maillard reactions occurring during the roasting process are reported to significantly affect the overall aroma and color characteristics (Murkovic and Derler 2006). Proteins also participate in these reactions by forming melanoidins and low-molecular-weight volatile compounds (Montavon et al. 2003). Quinic, citric, and malic acids contribute to the acidity of the beverage (Koshiro et al. 2015), whereas chlorogenic acids are mainly related to bitterness (Schenker and Rothgeb, 2017). Caffeine, a heat-stable compound, is also associated to bitterness (da Rosa et al. 2016). In addition, during the roasting process, lipids are prone to decomposition and auto-oxidation reactions, contributing to both aroma and body of the beverage (Koskei et al. 2015).

In recent years, consumers are demanding for high-quality coffee beverages, mainly considering their sensory characteristics. Among the many techniques available to assess the quality and composition of coffee, near-infrared spectroscopy (NIRS) has been widely used (Barbin et al. 2014). However, difficulties in the preparation of analytical curves and the cost of the equipment still prevent more application of NIRS. Therefore, in the last years, metabolomics has emerged as a potential tool for food quality and traceability (Senizza et al. 2019; Rocchetti et al. 2020). Accordingly, metabolomics has been applied to study different aspects of coffee processing. For example, the metabolomic profile of coffee was found to be affected by processing, roasting, grinding, as well as brewing methods (Selmar et al. 2006). In other studies, metabolomics-based approaches have been used to authenticate coffee (Jumhawan et al. 2016) or to discriminate coffee brewed by different methods (Xu et al. 2019). Regardless the brewing method chosen, the quality and sensorial attributes of coffee beans has a pivotal importance to determine the attributes that both tasters and consumers perceive from a sensorial point of view. Also, as reported by de Souza Gois Barbosa et al. (2019), comprehensive profiling of crude coffee can be indicative of the sensory quality of the coffee brews, thus providing relevant information to both producers and the coffee industry.

To the best of our knowledge, few studies in the literature tried to correlate the chemical composition of ground coffee to its sensorial quality. These kinds of studies are based on the targeted identification of possible quality markers. Therefore, in this study, we aimed to identify a consistent group of chemical markers that allow correlating the chemical profiles of ground coffee with its sensorial scores. To this aim, we used a combination of mass spectrometry techniques (both UHPLC-QTOF-MS and HS-GC-MS) followed by multivariate statistical modelling (both unsupervised and supervised approaches).

## Materials and methods

### Coffee samples and sensory analysis

Samples consisted of ground coffees obtained from the 2018 edition of the “International coffee tasting” (ICT). The latter is an international competition between coffee of a single origin or in mixture, in grains, in powder or single dose, considering the espresso, moka, or filter preparations. In this regard, 320 total samples from different countries (supplementary Table 1) have been accepted for the competition and analysed by five professional commissions (A-B-C-D-E). The commissions were balanced by sex, number, and origin of the judges (supplementary Table 1); each commission analysed an average of 42 samples (including replicates) according to an established schedule and using the “International coffee tasting” card on electronic support (supplementary Table 1). The sensory evaluations were then carried out using the Trialtest method and according to the characteristic phases of the sensory analysis, starting from the visual analysis, then olfactory, tactile taste, and the evaluation of the retro-olfactory perceptions.

Regarding the ground coffee samples used for chemical analyses, they were sampled during the competition that included products from a different origin, processing, and characteristics. The samples used were those that obtained either the higher (receiving a gold medal) and lower overall sensorial scores (not winning any gold medal by the panel of experts) in the competition. Further description of the 47 samples taken for experimentation, together with their random three digits code and sensory descriptors scores, can be found in supplementary Table 1. Overall, 20 coffee samples received a gold medal and classified as “high quality” group, while the remaining 27 samples did not receive any medal, thus being classified as “low quality” group.

### Extraction and UHPLC-QTOF-MS profiling of ground coffee

Three replicates (1 g) of each ground sample were extracted using an Ultra-Turrax homogenizer (IKA T25, Staufen, Germany) in 10 mL of 80% methanol solution (LC-MS grade, VWR, Milan, Italy) acidified with 0.1% formic acid. The extracts were then centrifuged (Eppendorf 5810R, Hamburg, Germany) at 10,000 × *g* for 10 min at 4 °C and filtered using 0.22 μm cellulose syringe filters into amber vials for further use.

The comprehensive chemical profile of ground coffee extracts was then investigated through an untargeted metabolomics-based approach (UHPLC-QTOF-mass spectrometry). In particular, a 1290 liquid chromatograph was coupled with a G6550 mass spectrometer detector via a Dual Electrospray Jet Stream ionization system (from Agilent Technologies, Santa Clara, CA, USA), under previously optimized instrumental conditions (Rocchetti et al. 2018). The instrument worked in positive polarity to acquire ions in SCAN mode (considering a range of 100–1200 m/z). Samples were acquired in “extended dynamic range” mode with a nominal resolution of 40,000 FWHM. The injection volume was 6 μL, in triplicate for each sample; the sequence injection was randomized and Quality Control samples (made by pooling an aliquot of each extract belonging to “high” and “low” groups) were injected at the beginning of the sequence and every 10 sample injections. Quality controls were acquired under the same conditions applied for samples, except for MS. In detail, QCs were analyzed in data-dependent MS/MS mode using 12 precursors per cycle (1 Hz, 50–1200 m/z, positive polarity, active exclusion after 2 spectra), with collision energies of 10, 20 and 40 eV for collision-induced decomposition.

### Post-acquisition data processing following UHPLC-QTOF-MS

The raw data were aligned and deconvoluted using the Agilent Profinder B.07 software. In this regard, the find-by-formula algorithm was used to annotate molecular features (MFs) following mass and retention time alignment. The minimum absolute abundance was set to 5000 counts, the mass accuracy was 5 ppm and the isotope model of “common organic molecules” was adopted. Only those molecular features present in at least 100% of one sample groups were considered as significant for further analysis. Overall, the MFE algorithm can locate multiple compounds within a single chromatographic peak and generates a list of MFs consisting of retention times (RT) and molecular masses. Thereafter, a list of possible molecular formulae was provided by considering both accurate monoisotopic masses (mass error ≤ 5 ppm) and isotopic patterns (i.e., isotopic distribution, space, and abundance). These latter were compared to those reported in three databases, namely the FoodDB (https://foodb.ca/- using the list of compounds already reported in coffee), Phenol-Explorer 3.6 (http://phenol-explorer.eu/- to profile polyphenols), and a custom database on Maillard reaction product (considering data reported in the literature). Features were aligned (mass tolerance window: 5 ppm + 2mDa; retention time tolerance: 0.15 min), and a post-acquisition filtering-by-frequency process was also adopted, to retain features present in 50% of replications within at least one treatment. Finally, abundances were log2 transformed and centered on the median of the individual features in the dataset. In our analytical conditions, a Level 2 of confidence (i.e., putatively annotated compounds) was achieved, as reported by COSMOS Metabolomics Standards Initiative (Salek et al. 2013). Also, to achieve a higher degree of confidence in annotation, the quality controls containing MS/MS spectra were elaborated in MS-DIAL (version 4.24) for a further identification/confirmation step (Tsugawa et al. 2015). To this aim, publicly available MS/MS experimental spectra built in the software (e.g., MoNA) and MS-Finder *in-silico* fragmentation from compounds in Lipid Maps, FoodDB, and PlantCyc (Tsugawa et al. 2016) were used.

Finally, the polyphenols annotated were also quantified by using standard methanolic solutions of compounds representative of their specific classes, namely cyanidin (anthocyanins), luteolin (flavones), catechin (flavonols), 5-pentadecylresorcinol (alkylphenols), tyrosols (low molecular weight phenolics) and chlorogenic acid (phenolic acids). In this regard, a linear fitting (not weighted and not forced to the origin) was built, expressing the results as mg/g phenolic equivalents (Rocchetti et al. 2019).

### Headspace/gas chromatography–mass spectrometry analysis of volatile compounds

Each coffee ground sample was then analyzed by HS–SPME–GC–MS using a CAR/PDMS fiber (100 μm) and a Rtx-5MS capillary column (30 m × 0.25 mm i.d., 0.25 µm film thickness; Restek Corporation, Bellefonte, PA, USA). GC–MS analysis was carried out using a TraceGQ Ultra (Thermo-Fisher Scientific, San Jose, CA, USA) coupled with an HS-SPME system (Triplus autosampler, Thermo-Fisher) and single quadrupole mass spectrometry (ISQ, ThermoFisher). Helium was the carrier gas with a column head pressure of 55 kPa. The conditions used for the HS-SPME study were as follows: incubation time 15 min, temperature 60 °C, extraction time 15 min and desorption time 6 min. The oven program was 40 °C for 5 min, with a rate of 5 °C/min up to 190 °C, maintained 8 min and up to 240 °C with a rate of 10 °C/min, maintained 10 min. The acquisition was in SCAN mode (35–350 m*/z*). The putative identification of volatile compounds was carried out using the NIST Chemistry WebBook spectrum library present in the equipment software.

### Multivariate statistical analysis

The normalization of metabolomics-based data was done using the Agilent Mass Profiler Professional B.12.06 software, as previously reported (Rocchetti et al. 2019). Afterward, an unsupervised hierarchical cluster analysis (HCA - distance measure: Euclidean; clustering algorithm: Ward’s) was produced on the normalized molecular features (MFs) by using the online software MetaboAnalyst (Xia et al. 2013). After that, supervised orthogonal partial least squares discriminant analyses (OPLS-DA) were carried out using SIMCA 13 software (Umetrics, Malmo, Sweden). In particular, different models were created from the metabolomics datasets containing (i) MFs (from MFE), (ii) typical compounds reported in coffee (from FoodDB), (iii) polyphenols (from Phenol-Explorer) and (iv) a combination of volatile and compounds and Maillard reaction products. Each OPLS-DA model built was then inspected for outliers, cross-validated (CV-ANOVA) and inspected for overfitting (permutation testing, N = 200) as previously described (Rocchetti et al. 2019). Thereafter, model parameters (R^2^Y and Q^2^Y) were recorded, and misclassification tables were generated. The variables importance in projection (VIP) was then used to select those compounds having the highest discrimination potential (VIP score > 0.8 - Senizza et al. 2020).

Moreover, the Receiver Operating Characteristics (ROC) curve approach was applied using Metabo Analyst (Xia et al. 2013) to validate the potential discriminant markers outlined by the VIP approach. Therein, the area under the ROC curve (AUC) was inspected to evaluate the global performance of each VIP marker. Finally, Pearson's correlations (p < 0.05, two-tailed) and principal component analysis (PCA) were performed using PASW Statistics 26.0. to correlate VIP markers (from OPLS-DA models) and sensory descriptors (from sensory analysis; supplementary Table 1).

## Results and discussion

### Untargeted screening by UHPLC-QTOF mass spectrometry of ground coffee samples

In this work, the untargeted annotation workflow based on UHPLC-QTOF-MS was able to reveal a total of 3002 MFs (supplementary Table 2), thus confirming the chemical complexity of the matrix under investigation. Thereafter, the unsupervised HCA was carried out in order to naïvely group the different ground coffee samples according to intrinsic similarities in their measurements (i.e., the different abundance of each annotated MF). In particular, each sample was characterized by a distinct up/down-accumulated cluster of compounds (supplementary Figure 1). The heat map showed three distinct subclusters, with some overlapping between "high" and “low” quality samples. This was somehow expected, considering that the sensory score is a continuous variable and that several other parameters (including confounding factors such as origin, cultivar, roasting) were present. Therefore, supervised statistics were next used to discriminate the ground coffee samples according to the overall sensory score. In particular, the multivariate supervised OPLS-DA statistical approach was used to provide the distribution of the different samples into the score plot hyperspace. Among the supervised methods, the OPLS-DA can consider only the Y-predictive variation, eliminating that not directly correlated with Y in the data matrix. As can be observed from supplementary Figure 2, the class prediction model discriminated the different samples, outlining a clear separation between “high” and “low” quality ground coffee replications and when considering the second latent vector. The OPLS-DA score plot built considering the overall sensorial score (i.e., “high” *vs.* “low”) as the discrimination factor is reported as supplementary Figure 1. As can be observed, coffee sample replicates were separated according to the class membership used; interestingly, this model also revealed very interesting information. In fact, ground coffee samples presenting the highest global score (i.e., C057; supplementary Table 1) were found to be located in a different area of the score plot hyperspace, thus confirming a distinctive chemical profile when compared to the other samples. Therefore, the chemometric approach clearly showed possible correlations between the two sample groups, quite representative of the differences revealed by the sensorial analysis by looking at the overall scores. Besides, in our experimental conditions, the model cross-validation parameters were excellent, being R^2^Y (goodness of fit) = 0.99 and Q^2^ (goodness of prediction) = 0.91. Also, the prediction model was found to possess an adequate CV-ANOVA coefficient (p < 0.001) and permutation test cross-validation (N = 200).

### Discrimination of coffee samples according to FoodDB and phenol-explorer databases

Considering that the main differences were actually represented in the metabolomics dataset, the following analysis was based on FoodDB (specifically considering the coffee composition) and Phenol-Explorer (for phenolics) as references for annotation.

**Fig. 1:**
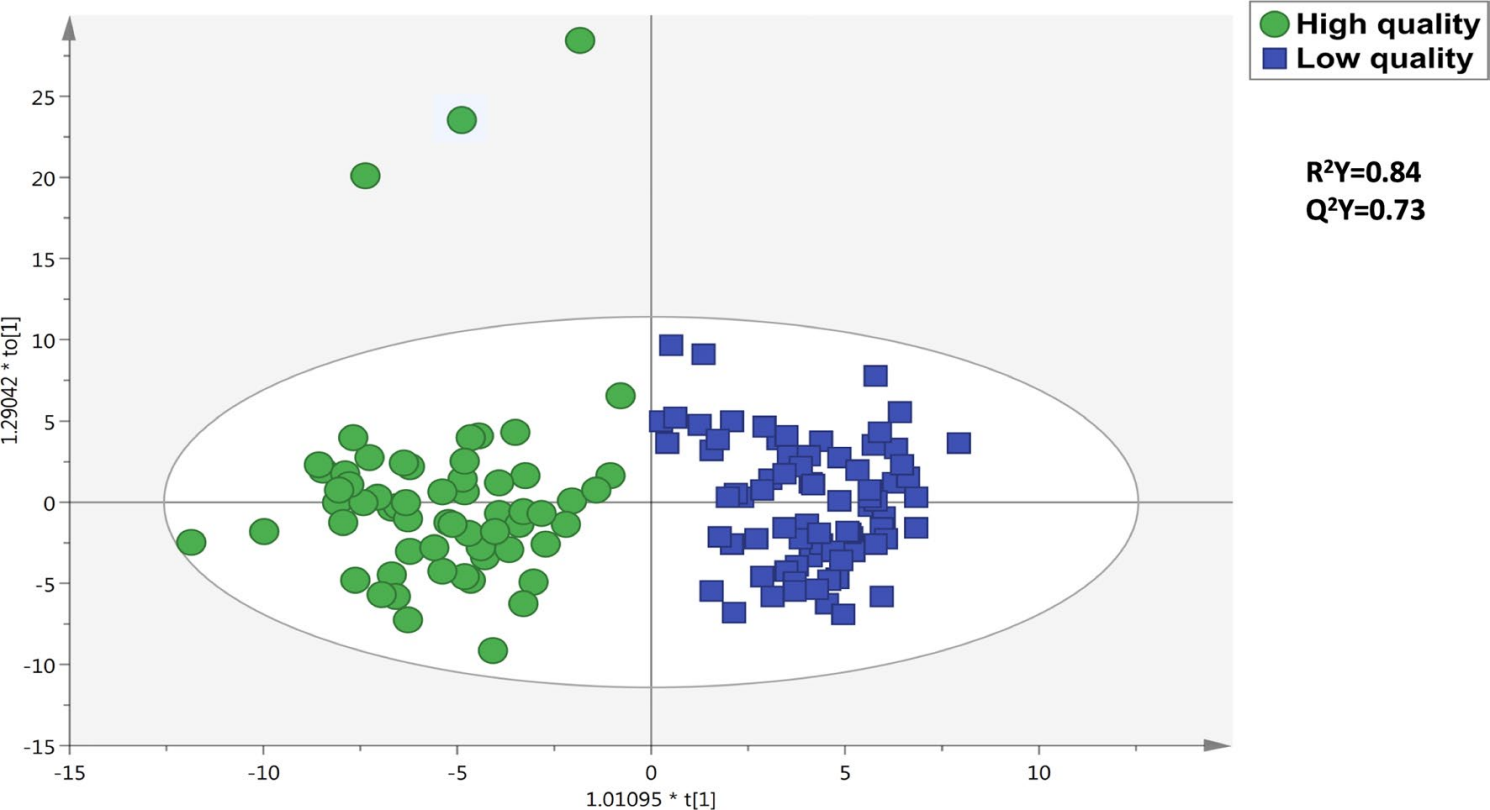
Orthogonal Projections to Latent Structures Discriminant Analysis (OPLS-DA) score plot for high *vs* low quality coffee samples. The output was built considering the annotations from FoodDB (UHPLC-QTOF-MS). Besides, the R^2^Y and Q^2^Y predictive parameters are also reported

First of all, we focused on the compounds annotated through FoodDB. As can be observed from supplementary Table 2, this approach allowed us to putatively annotate 338 compounds, according to the typical composition of coffee and coffee products (http://foodb.ca/). Besides, the dedicated tandem-MS approach using quality controls for each condition allowed us to record 96 unique structures. In particular, the identity of some metabolites was confirmed, including caffeine, trigonelline, 3-ethylpyridine, 1-*O*-caffeoylquinic, and 5-*O*-caffeoylquinic acids (supplementary Table 2). As expected, two of the major alkaloids, namely trigonelline (a pyridine alkaloid) and caffeine (1,3,7-Trimethylxanthine - a purine alkaloid), were successfully annotated and recorded very high abundance values (supplementary Table 2). In addition, other highly abundant compounds belonged to ketones, polyphenols, and pyrazines (supplementary Table 2). Caffeine is considered a key component in coffee sensorial quality, being related to several flavor attributes such as strength, body and bitterness. Besides, caffeine is quite stable during roasting conditions (Cheng et al. 2016). Additionally, trigonelline has been related to overall aromatic and bitterness perceptions; however, this pyridine alkaloid is reported to be quite unstable during roasting, being degraded, on average, by 60–90% (Cheng et al. 2016). Nevertheless, coffee quality is the final result of the interaction among different factors, mainly including genotype *x* environment interactions. In this regard, there is vast evidence and a common consensus about the strong influence of the interaction between genetic background and environment on the chemical phenotype of a plant, a phenomenon also known as phenotype plasticity (Lucini et al. 2020). In light of this, several other compounds were expected to specifically complement the sensorial traits of the coffees we analyzed. To detect the discriminant and potential marker compounds mainly responsible for the quality differences between the two sample groups, supervised multivariate statistics based on OPLS-DA modelling were applied. The OPLS-DA score plot built using FoodDB data is reported in Fig. 1. The model parameters were satisfying, being R^2^Y = 0.84 and Q^2^Y = 0.73. Besides, Fig. 1 indicates that the degree of discrimination was not so clear, likely indicating that the chemical distance between some coffee samples included in the “low quality” and some characterizing the "high quality" groups was shaded. This finding is consistent with the outcomes of sensory analysis (supplementary Table 1), where a continuum was recorded for sensory scores. Nonetheless, we found again that a completely distinctive chemical profile characterized C057 sample, compared to the others, thus strengthening the results of sensory analysis. Therefore, the next step was based on the identification of the most important variables in projection (VIP markers), i.e., those compounds determining the output observed in Fig. 1. The discriminant compounds allowing grouping are reported in Table 1, together with their VIP scores and Log Fold-Change values (resulting from Fold-Change analysis with cut-off = 2 and having a p-value < 0.05). Overall, we classified 37 discriminant compounds showing intriguing differences between “high” *vs*. “low” quality coffee samples. In this regard, 24% of VIP markers was down-accumulated in “high” quality samples, while the remaining 76% was found to be up-accumulated. Therefore, this approach suggests that “high quality” ground coffee samples were characterized by the presence of compounds likely responsible for the different global scores obtained during the sensory analysis. Going into the details, those compounds possessing the highest down-accumulation trends were 2-Ethyl-5-methylpyridine (LogFC = −2.97), followed by 4-[(2-Furanylmethyl)thio]-2-pentanone (LogFC = −2.94), and (R)-2,7-Dihydroxy-2H-1,4-benzoxazin-3(4H)-one 2-glucoside (LogFC = −2.69). Regarding the up-accumulated compounds, we found a significant abundance of mascaroside (VIP score = 1.32 and Log FC = 6.87) in high-quality coffee samples (Table 2), followed by 1-(Methylthio)-2-butanone (VIP score = 1.03 and LogFC = 5.78) and isomeric forms of diazines (VIP score = 1.40 and LogFC = 4.60). It is also important to notice that isomeric forms of chlorogenic acids were classified as discriminant in the model and particularly abundant in “high-quality” samples (VIP score = 1.10; Log FC = 0.89). The chlorogenic acids are known to be responsible for astringency, coffee pigmentation, and aroma formation (Kreuml et al. 2013). Furthermore, their thermal degradation during the roasting process also contributes to bitterness (Kreuml et al. 2013).

**Table 1:** Variables importance in projection following OPLS-DA modelling, considering the annotations from FoodDB (UHPLC-QTOF-MS)

Class (FooDB)	VIP marker (OPLS-DA)	VIP score (OPLS-DA)	Log2 Fold-Change (High *vs* Low quality)	ROC AUC
Azoles	5-Acetyl-2,4-dimethyloxazole	1.11 ± 0.39	−1.13	0.64
Benzene and substituted derivatives	2/3/4-Methylbenzaldehyde	1.63 ± 0.32	−1.58	0.77
	4-Ethyl-1,2-dimethoxybenzene	1.09 ± 0.83	0.44	0.55
	Styrene	1.27 ± 0.49	0.69	0.69
Carboxylic acids and derivatives	3-Mercapto-3-methylbutyl formate	1.82 ± 0.37	1.45	0.73
	L-Homoserine	1.86 ± 0.39	1.73	0.74
Diazanaphthalenes	5-Methylquinoxaline	1.19 ± 0.58	0.50	0.67
Diazines	Propylpyrazine/Trimethylpyrazine/2-Ethyl-5-methylpyrazine, 9CI, 8CI/2-Ethyl-6-methylpyrazine	1.40 ± 0.46	4.60	0.63
	2,5-Diethyl-3-methylpyrazine/3,5-Diethyl-2-methylpyrazine/2-Methyl-3-(2-methylpropyl)pyrazine	1.92 ± 0.50	2.30	0.79
Fatty Acyls	1-(3-Methylbutanoyl)-6-apiosylglucose	1.10 ± 0.65	1.24	0.62
	Methyl pentanoate	1.73 ± 0.65	0.96	0.78
	Methyl hexanoate	1.83 ± 0.32	0.53	0.73
Flavonoids	Scorzonoside	1.12 ± 1.16	−0.64	0.52
Heteroaromatic compounds	2-(Methoxymethyl)furan/3-Methyl-1,2-cyclopentanedione	1.17 ± 0.53	−0.85	0.73
	3-(2-Furanyl)-2-propenal	1.00 ± 0.53	1.33	0.65
	4-[(2-Furanylmethyl)thio]-2-pentanone	1.04 ± 0.35	−2.94	0.66
	2-[(Ethylthio)methyl]furan/2,5-Dimethyl-3-(methylthio)furan	1.63 ± 0.23	1.93	0.67
Imidazopyrimidines	Caffeine	1.10 ± 0.36	−1.13	0.52
Naphthofurans	Mascaroside	1.32 ± 1.00	6.87	0.58
Organooxygen compounds	(R)-2,7-Dihydroxy-2H-1,4-benzoxazin-3(4H)-one 2-glucoside	1.21 ± 0.80	−2.69	0.71
	Torachrysone 8-(2-apiosylglucoside)	1.67 ± 0.82	1.69	0.53
	alpha-Furyl methyl diketone	1.82 ± 0.43	−1.73	0.76
	Tryptophol [xylosyl-(1-6)-glucoside]	1.03 ± 0.45	1.76	0.61
	1-(Methylthio)-2-butanone	1.03 ± 0.96	5.78	0.67
	Coniferin	1.07 ± 0.48	1.30	0.59
	1-Phenyl-1-propanone	1.52 ± 0.36	1.42	0.79
	Thalictroidine	1.73 ± 0.70	2.63	0.72
	2-Acetyl-5-methylthiophene/2-(Methylthio)phenol/Kahweofuran	2.01 ± 0.66	1.23	0.78
	4-Acetyl-2-methylpyridine	2.05 ± 0.17	1.65	0.84
Phenolic acids	trans-Chlorogenic acid/trans-Neochlorogenic acid	1.10 ± 0.55	0.89	0.57
Phenols	4-Ethyl-2-methylphenol/2-Ethyl-4-methylphenol	1.30 ± 0.79	4.10	0.59
	erythro-Syringoylglycerol	1.86 ± 0.27	0.43	0.69
Pyridine and derivatives	2-Ethyl-5-methylpyridine	1.07 ± 0.28	−2.97	0.62
Tetrahydroisoquinolines	*O*-Methylcorypalline	2.18 ± 0.34	0.78	0.79
Thioethers	Methyl phenyl sulfide	1.01 ± 0.39	1.70	0.53
Tropane alkaloids	Calystegine A6	1.07 ± 0.85	2.05	0.56
	Calystegine C1	1.14 ± 0.39	0.32	0.61

Each compound is reported according to its chemical class, together with VIP score, Log2 FC value, and ROC AUC value

**Table 2:** Variables importance in projection following OPLS-DA modelling, considering the annotations based on the volatile compounds (Head Space-GC-MS) and Maillard reaction products (UHPLC-QTOF-MS)

Class	VIP marker (OPLS-DA)	Description ^a^	VIP score (OPLS-DA)	Log2 FC (High vs Low quality)	ROC AUC
Heteroaromatic compounds	2-Furfurylthiol^b^	Roasted, bitter	1.90 ± 0.87	0.57	0.75
Phenols	Guaiacol^b^	Phenolic, burnt, smoky	1.37 ± 0.70	0.19	0.65
Heteroaromatic compounds	Furfuryl methyl ether^**b**^	Savory	1.36 ± 0.52	0.59	0.59
Pyrroles	1-Furfurylpyrrole^b^	Cocoa, green, roasted	1.33 ± 0.64	0.35	0.58
Organooxygen compounds	Hexanal^b^	Green, grassy, fruit	1.32 ± 0.49	0.27	0.58
Phenols	4-Ethyl-2-methoxyphenol^b^	Clove, phenolic, spice	1.32 ± 0.60	1.28	0.69
Carboxylic acids and derivatives	3-Mercapto-3-methylbutyl formate^b^	Roasted	1.32 ± 0.84	−1.10	0.51
Phenols	*p*-Vinylguaiacol^b^	Clove, curry, spice	1.10 ± 0.71	−0.35	0.57
Organooxygen compounds	Acetylpropionyl^b^	Buttery	1.05 ± 0.64	−1.80	0.68
Glucose degradation products	3,4-Dideoxyglucosone^b^	Maillard reaction	1.03 ± 0.51	−0.50	0.51
Lactones	2-methyltetrahydrofuran-3-one^b^	Nuts	1.00 ± 0.88	−0.88	0.67
Pyridines and derivatives	Pyridine^c^	Sour, putrid, bitter, roasted	0.94 ± 0.59	−0.13	0.62
Organooxygen compounds	2-Methylpropanal^c^	Aldehydic, floral, green	0.94 ± 0.51	−1.19	0.63
Diazines	2-Ethyl-3-methylpyrazine^c^	Nutty, peanut	0.90 ± 0.53	−0.32	0.53
Advanced glycation end-product	N-(carboxymethyl)lysine^b^	Maillard reaction	0.90 ± 1.09	0.83	0.59
Organooxygen compounds	2-Methylbutanal^c^	Chocolate, caramel, nutty	0.89 ± 0.42	−0.50	0.61
Diazines	2-Ethyl-6-methylpyrazine^c^	Flowery, fruity, hazelnut	0.88 ± 0.13	−0.25	0.55
Glucose degradation products	3-Deoxyglucosone^b^	Maillard reaction	0.82 ± 0.67	0.21	0.50

Each compound is reported according to its chemical class, together with VIP score, Log2 FC value, and ROC AUC value

^a^Sensory descriptors reported according to previous works (Yang et al. 2016)

^b^Identify by HS-GC-MS

^c^identify by UHPLC-QTOF-MS

After that, a third OPLS-DA predictive model was built considering the polyphenols annotated in both coffee sample groups, according to the database Phenol-Explorer. Each (poly)-phenolic compound annotated in our experimental conditions is reported in supplementary Table 2, grouped in the corresponding class and sub-class. By using this approach, 80 compounds were detected, being 45 flavonoids (mainly flavone equivalents), 17 lower-molecular-weight phenolics (tyrosol equivalents), 14 phenolic acids (mainly hydroxycinnamics), and 4 alkylphenols. The phenolic composition was the characteristic of coffee, with a large abundance of isomeric forms of caffeoylquinic acid, alkylphenols (such as 4-methylcatechol), methoxyphenols (i.e., guaiacol), and other polyphenols (catechol). Also, a semi-quantitative analysis (based on representative phenolic standard compounds) allowed grouping and quantifying each phenolic class separately. The results are reported in supplementary table 3. Overall, the total phenolic content ranged from 14.2 mg/g in C321 sample (low quality) up to 37 mg/g in C057 (i.e., the sample characterized by the highest overall sensory score). The phenolic dataset was then evaluated to discriminate coffee samples according to their sensory quality using OPLS-DA. The score plot (supplementary table 3) indicated that phenolic signatures were not good in providing adequate discrimination (goodness parameters of the model: R^2^Y = 0.63 and Q^2^Y = 0.50). Therein, isomeric forms of flavones, flavonols, and chlorogenic acids showed the highest discrimination potential (supplementary table 3). Nonetheless, polyphenols were not among the main contributors to the global sensory scores obtained.

### Discrimination of coffee samples according to volatile compounds and maillard reaction products

**Fig. 2:**
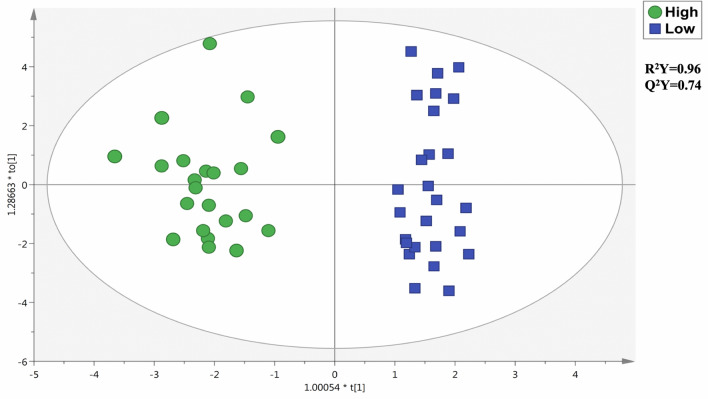
Orthogonal Projections to Latent Structures Discriminant Analysis (OPLS-DA) score plot for high *vs* low quality coffee samples. The output was built considering the annotations based on the volatile compounds (Head Space-GC-MS) and Maillard reaction products (UHPLC-QTOF-MS). Besides, the R^2^Y and Q^2^Y predictive parameters are also reported

The latter approach was based on the identification of volatile compounds by using Head Space-GC-MS and Maillard reaction products from UHPLC-QTOF-MS analysis. The datasets obtained were then combined to perform multivariate statistical modelling. Overall, we found an adequate degree of discrimination according to the sensory scores, as provided in Fig. 2. In particular, the OPLS-DA model was characterized by more than reliable goodness parameters, being R^2^Y = 0.96 and Q^2^Y = 0.74. The following VIP approach allowed us to list the most discriminant compounds (high *vs.* low quality). The VIP markers obtained are reported in Table 2, together with their sensory descriptors, class, VIP scores, and Log Fold-Change values. Interestingly, 18 compounds (including both volatiles and Maillard reaction products) possessed a VIP score higher than 0.8. However, 55.6% of the discriminant markers were down accumulated in high-quality coffee samples when compared with the other sample group. The VIP approach combined with the ROC curve inspection, also showed that 2-Furfurylthiol was the Maillard reaction product characterized by the highest discrimination potential, possessing a VIP score = 1.90 and a ROC AUC value = 0.75. Also, guaiacol (VIP score = 1.37) and furfuryl methyl ether (VIP score = 1.36) were found to be up accumulated in high-quality samples, although the ROC curves outlined scarce potentials as discriminant markers. Besides, among the volatile compounds detected by HS-GC/MS, we found 5 compounds: pyridine, 2-Methylpropanal, 2-Ethyl-3-methylpyrazine, 2-Methylbutanal, and 2-Ethyl-6-methylpyrazine (Table2 ). Therefore, our experimental workflow suggested that volatile compounds and Maillard reaction products, rather than phenolic compounds, were better correlated with the sensory quality scores recorded during the international coffee tasting.

According to the literature, aldehydes, ketones, alcohols, esters, pyrazines, furans, and other compounds are among the most important volatile fraction characterizing roasted coffee (Caporaso et al. 2018). In particular, the chemical processes affecting the development of volatile compounds in coffee are mainly related to Maillard or non-enzymatic browning, Strecker degradation, degradation of individual amino acids, trigonelline, sugars, polyphenols (mainly phenolic acids), lipids, and the interactions between all the intermediate decomposition products. In previous work, de Toledo et al. (2017) used the volatile fraction to build statistical models for the discrimination of coffee geographical origins. In this regard, 2-methylpyrazine and pyridine were reported as the most effective compounds for the discrimination of coffee geographical origins, explaining 97.3% of the variance. In addition, Casas and co-authors (2017) identified some potential markers of defective *Coffea arabica* L. beans, showing that some compounds, such as 1-methylpyrrole, 5-methyl-2-furfurylfuran, and 2-methylfuran, were uniquely present in defective fractions. Overall, that 35 compounds dominated by volatile compounds, organic acids, sugars and sugar alcohols were sufficient to separate the defective and non-defective fractions of roasted coffee beans.

### Correlations between discriminant markers and sensory data

**Fig. 3:**
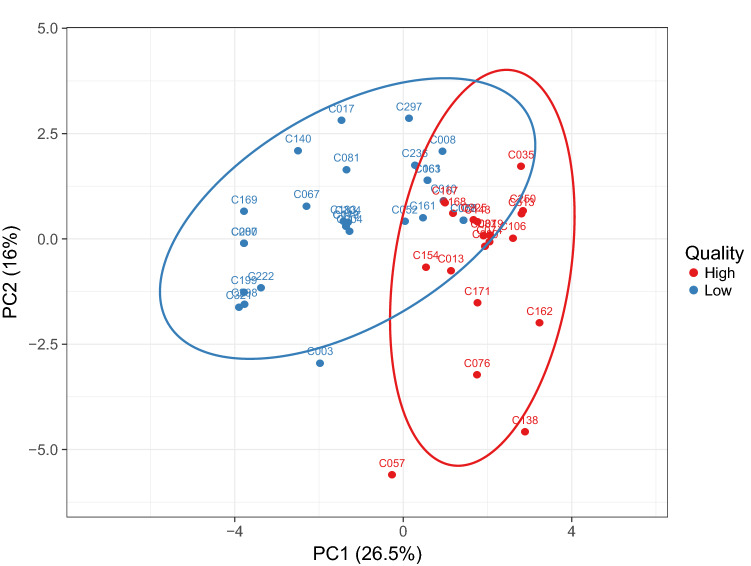
Principal component analysis (PCA) score plot obtained by considering the VIP marker compounds (Table 1 and Table 2) and sensory descriptor scores (supplementary material)

To correlate the VIP markers provided by the different prediction models (from OPLS-DA), Pearson’s correlation coefficients and unsupervised PCA then carried out. The PCA score plot obtained by considering both VIP markers (Tables 1 and 2) and sensory descriptors is reported in Fig. 3. As can be observed, the two principal components explained 26.5% and 16% of the total variance, respectively. In particular, partial discrimination was obtained between the two sample groups (i.e., high *vs.* low quality). Some samples belonging to the “low-quality” group (Fig. 3) possessed some analogies with samples classified as “high-quality”, thus likely affecting the sample group discrimination. Interestingly, the coffee sample receiving the highest global sensory score (i.e., C057; supplementary Table 1) was again found to possess the most characteristic profile. Afterward, Pearson's correlation coefficients were inspected in order to identify those discriminant compounds better correlated to the sensory quality and then better explaining the trends observed in the PCA score plot. Overall, methyl pentanoate, 2-furfurylthiol, L-Homoserine, and pyridine were those VIP markers that established the higher number of significant correlations (p < 0.05) when compared to the other compounds (supplementary table 4). In addition, we found that our chemical data were able to explain mainly the olfactory richness, with 28 compounds establishing significant correlations (p < 0.05). Looking to the most significant compounds, methyl pentanoate was significantly correlated to the tactile balance (0.66) and olfactory richness (0.55); this compound, also known as methyl valerate, is described as having a pungent-ethereal, green-fruity apple-like odor of poor tenacity. Regarding 2-furfurylthiol (or 2-furanmethanetiol), this compound is described as a powerful aromatic volatile thiol, reported to contribute to the aroma of roasted coffee. In our experimental conditions, we found this compound highly correlated to the tactile balance (0.75), followed by a hedonic (or total pleasure) level (0.58). However, it is important to underline that a global sensory perception (such as those related to the tactile balance and body attributes) are always the result of synesthetic and complex phenomena involving several sensory descriptors, rather than one compound, with synergies among stimuli, oral processing, and dynamic evolution of the sensory stimulus playing a major role in the aroma perception (Dulsat-Serra et al. 2016). The other compounds highly correlated to the sensory quality were pyridine and L-Homoserine. Pyridine is usually formed during long roasting conditions and has previously been proposed to originate during roasting through the decomposition of trigonelline and via the Maillard reaction processes. According to Pearson’s correlations (supplementary table 4), pyridine was particularly correlated (p < 0.05) to roasted (0.68) and bitterness (0.69) sensory descriptors. Our findings fitted with Yang et al. (2016), outlining pyridine as particularly related to bitter, astringent, roasted, and burnt descriptors. Regarding L-Homoserine, we found a high and significant correlation with “body” as a sensory descriptor (supplementary table 4). Homoserine, or its lactone form, is the product of a cyanogen bromide cleavage of a peptide by the degradation of methionine. To the best of our knowledge, no works have previously related this compound to the sensory quality of coffee and coffee products; therefore, further *ad-hoc* and targeted studies are deemed to confirm these findings.

## Conclusions

In this work, we aimed to evaluate the potential of metabolomics to discriminate ground coffees with different sensory quality, as reported by a panel of coffee tasters. In particular, the potential of untargeted UHPLC-QTOF mass spectrometry was tested, thus providing discrimination of 47 ground coffee samples collected during the “International Coffee Tasting” (ICT) competition (2018 Edition). Overall, coffee samples were classified into two main groups, being “high quality” (i.e., 20 samples received a gold medal during the competition) and “low quality” group (27 samples, without medal). The UHPLC-QTOF metabolomics followed by multivariate supervised/unsupervised statistics allowed us profiling several classes of compounds, such as polyphenols, lipids, alkaloids, diazines, and Maillard reaction products, whilst a Headspace coupled with GC-MS approach highlighted the most important volatile compounds as related to coffee quality. Our findings demonstrated that polyphenols were not fully correlated to the sensory scores, whilst significant correlations were found when considering typical coffee metabolites (from Food Database) together with the volatile/Maillard compounds. In this regard, the OPLS-DA prediction models built considering the previously reported compounds were characterized by the highest goodness and accuracy parameters, recording prediction values > 0.7. Interestingly, the coffee samples having the highest sensory scores were found to be characterized by specific metabolomic signatures, thus corroborating the results provided by the coffee tasters during the competition. Interestingly, we found that, among the variables with the highest discrimination potential, methyl pentanoate (VIP score = 1.73; ROC value = 0.78), 2-furfurylthiol (VIP score = 1.90; ROC value = 0.75), and L-Homoserine (VIP score = 1.86; ROC value = 0.74) were the most correlated (p < 0.05) with the sensory parameters. Therefore, our findings demonstrate the suitability of untargeted metabolomics to assess the coffee quality and its correlation to sensory perceptions.

## Electronic supplementary material

Below is the link to the electronic supplementary material.

Supplementary file1 (TIF 669 kb)

Supplementary file2 (PNG 539 kb)

Supplementary file3 (DOCX 382 kb)

Supplementary file4 (XLSX 3230 kb)

Supplementary file5 (XLSX 277 kb)

Supplementary file6 (XLSX 47 kb)

Supplementary file7 (DOCX 15 kb)

## Data Availability

The metabolomics and metadata reported in this paper are available as supplementary material.
