# Supplementary figures and images for: Identification of markers of sensory quality in ground coffee: an untargeted metabolomics approach
Source: Metabolomics. 2020 Dec 14;16(12):127. doi: 10.1007/s11306-020-01751-6 (PMC7736008; doi:10.1007/s11306-020-01751-6)

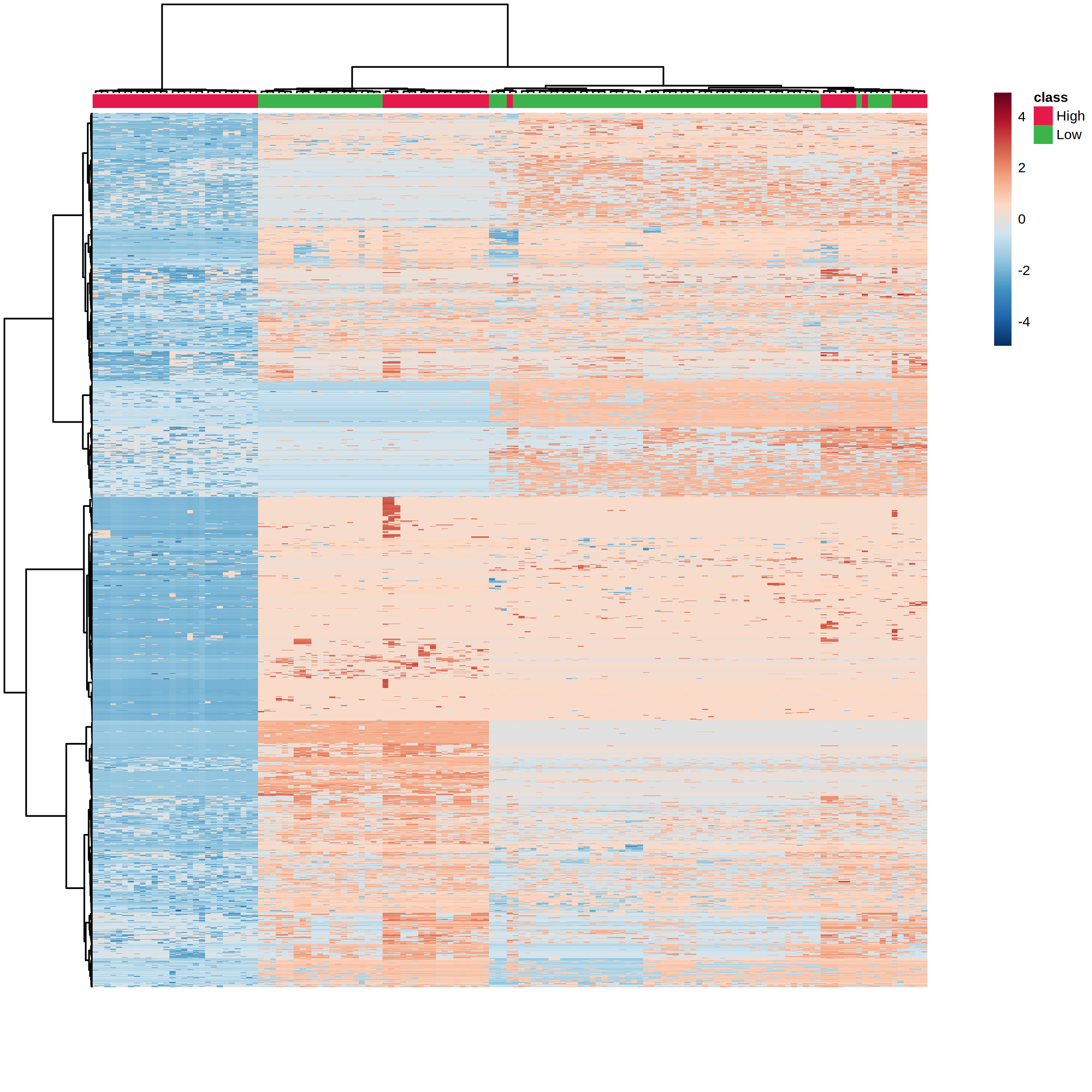

Supplement: Supplementary file 1 — Supplementary file1 (TIF 669 kb) [file 11306_2020_1751_MOESM1_ESM.tiff]

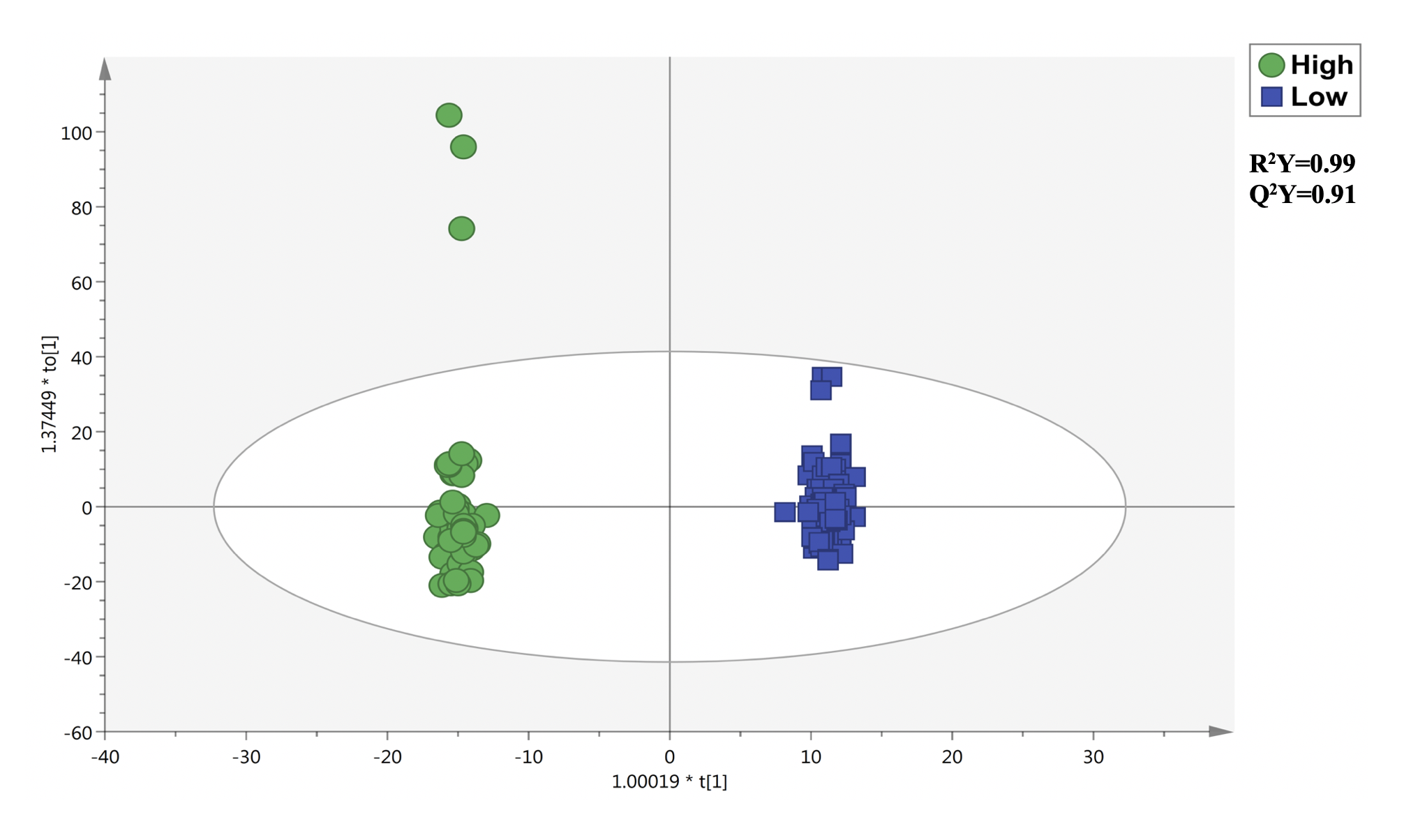

Supplement: Supplementary file 2 — Supplementary file2 (PNG 539 kb) [file 11306_2020_1751_MOESM2_ESM.png]
