# Supplementary material for: Identification of markers of sensory quality in ground coffee: an untargeted metabolomics approach
Source: Metabolomics. 2020 Dec 14;16(12):127. doi: 10.1007/s11306-020-01751-6 (PMC7736008; doi:10.1007/s11306-020-01751-6)
Supplement: Supplementary file 3 — Supplementary file3 (DOCX 382 kb) [file 11306_2020_1751_MOESM3_ESM.docx]

**Identification of markers of sensory quality in ground coffee: an untargeted metabolomics approach**

Gabriele Rocchetti, Gian Paolo Braceschi, Luigi Odello, Terenzio Bertuzzi, Marco Trevisan, Luigi Lucini

**Supplementary table 1**

Organized with the scientific support of the CSA ("Centro Studi Assaggiatori") Italian Tasters, the high quality of International Coffee Tasting (ICT) competition is based on the technical evaluations provided by the judges from the International Institute of Coffee Tasters (“Iiac”): a network that now counts almost 12,000 professionals in more than 40 countries around the world. The judges work completely blindfolded in accordance with the most stringent sensory analysis standards. All the data that they provide is statistically validated. Only products that pass the requirements established by the rules may receive a medal. The data used in this work were collected during the competition (November 2018). Overall, 320 total single origin or blends in grains samples were selected for the competition, as follows: 1 Australia, 5 Canada, 59 China, 4 Germany, 1 Greece, 134 Italy, 9 Japan, 15 Korea, 2 Peru, 2 Portugal, 5 Russia, 9 Slovenia, 11 Spain, 6 Switzerland, 40 Taiwan, 12 Thailand, 1 Netherlands, and 4 USA. Also, 30 professionally trained panelists (balanced for sex) from 10 different countries (such as Thailand, Italy, Japan, Korea, Bulgaria, Spain, Russia, USA, Germany, and Slovenia) participated in this study. Each panelist had >1200 h of experience in evaluating food and beverage products, including brewed coffee. The different coffee samples were prepared by professional operators, graduated in the "Espresso Italiano Specialist" course, and operating in compliance with the guidelines for the correct preparation according to the various methods required, namely filter, mocha, single-dose machines, and espresso bar machines. Also, random codes were assigned to the different coffee samples in order to ensure anonymity. The trained panelists evaluated an average of 42 samples on November 29th and 35 samples on November 30th, according to an established schedule. For the evaluation, they used the "International Coffee Tasting" form on electronic support, reporting the characteristic parameters of the sensory analysis starting from the visual (including colour intensity, texture, and attractiveness), then olfactory and retro-olfactory (including flowers and fresh fruit, vegetable, dried fruits and nuts, roasted, spicy, empyreumatic, biochemical, overall positive odors, overall negative odors, aroma persistence, finesse, richness, and hedonic level), gustatory and tactile (including body, acidity, bitterness, astringent, gustative balance, and tactile balance). The sensory evaluations were carried out using the "Trialtest method"; this method allows first to identify a descriptive profile of the product and then to determine both its quantitative and qualitative profiles. In addition, this test allows the validation of the results considering 3 different indicators: 1) control of the reliability of the descriptors (cut-off ≥ 6); 2) control of the index of effectiveness (reliability and quality) of the judges; 3) control of the form used to measure the sensory values. Overall, the result about the reliability of the quantitative descriptors is summarized in a global reliability index, inserted in the same table, calculated as % of the descriptors with reliability ≥ 6 when compared with the total provided by each descriptor. Finally, the ground coffee samples used for the metabolomic analysis were those obtaining either the higher (> 70; receiving a gold medal) and lower overall sensorial scores (< 70; not receiving any gold medal by the panel of experts) in the competition.

**Assessment form.**

**Coffee samples used for the trial.**

| ***Codice*** | ***Nazione*** | ***Categoria*** | ***COLOUR INTENSITY*** | ***TEXTURE*** | ***OLFACTORY INTENSITY*** | ***BODY*** | ***ACIDITY*** | ***BITTERNESS*** | ***ASTRINGENT*** | ***FLOWERS AND FRESH FRUIT*** | ***VEGETABLE*** | ***DRIED FRUITS AND NUTS*** | ***ROASTED*** | ***SPICY*** | ***EMPYREUMATIC*** | ***BIOCHEMICAL*** | ***OVERALL POSITIVE ODOURS*** | ***OVERALL NEGATIVE ODOURS*** | ***AROMA PERSISTENCE*** | ***Attractiveness*** | ***Olfactory richness*** | ***Olfactory frankness*** | ***Gustative balance*** | ***Tactile balance*** | ***Finesse*** | ***Richness*** | ***HEDONIC LEVEL*** | ***Punteggio*** | ***Punteggio 100*** |
| --- | --- | --- | --- | --- | --- | --- | --- | --- | --- | --- | --- | --- | --- | --- | --- | --- | --- | --- | --- | --- | --- | --- | --- | --- | --- | --- | --- | --- | --- |
| C000 | Italy | E | 7,00 | 6,50 | 7,50 | 5,50 | 2,00 | 4,00 | 1,00 | 2,50 | 1,50 | 3,00 | 6,00 | 2,00 | 1,00 | 0,50 | 4,50 | 1,50 | 6,00 | 7,00 | 5,50 | 4,50 | 3,50 | 4,00 | 4,50 | 4,50 | 4,50 | 55,00 | 55,56 |
| C003 | España | F | 7,00 | 8,00 | 6,50 | 5,00 | 6,50 | 2,50 | 0,50 | 6,00 | 1,50 | 2,50 | 3,00 | 2,00 | 0,00 | 0,50 | 5,00 | 1,50 | 6,00 | 7,50 | 6,50 | 7,00 | 5,00 | 4,50 | 6,00 | 5,50 | 6,00 | 69,00 | 69,70 |
| C008 | Australia | F | 7,00 | 7,00 | 6,00 | 6,00 | 3,00 | 3,00 | 1,00 | 5,00 | 1,00 | 5,00 | 6,00 | 2,00 | 1,00 | 0,00 | 6,00 | 1,00 | 6,00 | 6,00 | 6,00 | 6,00 | 4,00 | 5,00 | 6,00 | 5,00 | 5,00 | 66,00 | 66,67 |
| C010 | Italy | E | 8,00 | 7,00 | 6,00 | 6,00 | 3,00 | 4,00 | 3,00 | 3,00 | 1,00 | 5,00 | 7,00 | 3,00 | 2,00 | 1,00 | 6,00 | 2,00 | 7,00 | 7,00 | 6,00 | 5,00 | 4,00 | 4,00 | 6,00 | 5,00 | 6,00 | 63,00 | 63,64 |
| C013 | Italy | E | 7,00 | 8,00 | 7,00 | 6,00 | 4,50 | 3,50 | 0,00 | 4,50 | 0,50 | 5,00 | 5,50 | 1,50 | 1,00 | 0,00 | 6,50 | 1,00 | 6,50 | 7,50 | 7,00 | 7,00 | 6,50 | 6,00 | 6,50 | 6,50 | 7,00 | 77,50 | 78,28 |
| C017 | Germany | F | 7,00 | 8,00 | 7,00 | 7,00 | 3,00 | 4,00 | 1,00 | 3,00 | 1,00 | 4,00 | 6,00 | 3,00 | 1,00 | 1,00 | 7,00 | 1,00 | 7,00 | 7,00 | 6,00 | 6,00 | 5,00 | 5,00 | 5,00 | 5,00 | 5,00 | 65,00 | 65,66 |
| C019 | Germany | F | 7,00 | 7,00 | 7,00 | 7,00 | 5,00 | 5,00 | 2,00 | 4,00 | 2,00 | 5,00 | 5,00 | 1,00 | 0,00 | 0,00 | 6,00 | 2,00 | 6,00 | 7,00 | 7,00 | 6,00 | 5,00 | 6,00 | 6,00 | 6,00 | 6,00 | 72,00 | 72,73 |
| C035 | Italy | E | 7,00 | 8,00 | 7,50 | 7,00 | 4,00 | 4,00 | 2,00 | 3,50 | 2,50 | 3,00 | 7,00 | 1,50 | 0,00 | 1,50 | 6,50 | 3,00 | 7,00 | 7,50 | 6,50 | 6,00 | 5,50 | 6,00 | 5,50 | 6,50 | 6,00 | 69,50 | 70,20 |
| C050 | Italy | E | 7,00 | 8,00 | 7,00 | 7,00 | 2,50 | 2,50 | 0,50 | 3,50 | 0,00 | 2,50 | 6,50 | 2,00 | 0,50 | 0,00 | 6,50 | 1,50 | 6,50 | 7,50 | 6,00 | 6,00 | 6,00 | 5,50 | 6,00 | 6,50 | 7,00 | 71,50 | 72,22 |
| C052 | Italy | E | 9,00 | 6,00 | 7,00 | 7,00 | 2,00 | 5,00 | 2,50 | 1,50 | 2,50 | 5,00 | 6,00 | 2,50 | 2,50 | 2,00 | 4,50 | 2,50 | 6,50 | 6,00 | 5,50 | 4,50 | 3,00 | 4,50 | 4,00 | 5,00 | 4,00 | 54,00 | 54,55 |
| C057 | Italy | E | 7,00 | 7,00 | 7,00 | 5,00 | 4,00 | 3,00 | 0,00 | 5,50 | 0,50 | 5,50 | 5,50 | 1,50 | 0,00 | 0,00 | 8,00 | 0,00 | 6,50 | 7,00 | 7,00 | 7,00 | 7,00 | 7,00 | 7,50 | 7,00 | 8,00 | 80,50 | 81,31 |
| C058 | Italy | E | 6,00 | 8,00 | 7,00 | 6,00 | 4,00 | 3,00 | 1,00 | 4,00 | 1,00 | 3,00 | 5,00 | 1,00 | 1,00 | 0,00 | 7,00 | 1,00 | 6,00 | 6,00 | 7,00 | 7,00 | 5,00 | 5,00 | 6,00 | 6,00 | 6,00 | 69,00 | 69,70 |
| C061 | Italy | E | 7,00 | 8,50 | 7,50 | 5,50 | 2,00 | 6,50 | 1,00 | 3,00 | 2,00 | 3,00 | 5,50 | 2,50 | 1,00 | 0,00 | 6,00 | 1,00 | 6,00 | 8,00 | 7,00 | 6,50 | 4,00 | 4,50 | 4,50 | 5,50 | 5,50 | 65,50 | 66,16 |
| C067 | Italy | E | 7,00 | 6,00 | 7,00 | 6,00 | 6,00 | 1,00 | 1,00 | 5,00 | 2,00 | 5,00 | 4,00 | 3,00 | 0,00 | 0,00 | 6,00 | 2,00 | 6,00 | 4,00 | 6,00 | 6,00 | 4,00 | 5,00 | 6,00 | 5,00 | 5,00 | 63,00 | 63,64 |
| C073 | Italy | E | 6,00 | 7,00 | 6,00 | 6,00 | 6,00 | 3,00 | 1,00 | 3,00 | 2,00 | 4,00 | 4,00 | 2,00 | 1,00 | 1,00 | 6,00 | 1,00 | 7,00 | 6,00 | 6,00 | 6,00 | 4,00 | 4,00 | 6,00 | 5,00 | 5,00 | 61,00 | 61,62 |
| C074 | Italy | E | 8,00 | 7,50 | 7,00 | 5,50 | 6,50 | 2,00 | 1,00 | 5,00 | 1,50 | 4,50 | 4,50 | 1,00 | 0,50 | 0,50 | 6,00 | 1,50 | 7,00 | 7,00 | 6,50 | 6,50 | 5,00 | 5,00 | 6,00 | 6,50 | 5,50 | 70,00 | 70,71 |
| C076 | Italy | E | 7,00 | 7,00 | 7,00 | 6,00 | 5,00 | 3,00 | 1,00 | 4,00 | 1,00 | 5,00 | 6,00 | 1,00 | 0,00 | 0,00 | 7,00 | 0,00 | 8,00 | 7,00 | 7,00 | 7,00 | 6,00 | 7,00 | 8,00 | 7,00 | 8,00 | 79,00 | 79,80 |
| C077 | Italy | E | 7,00 | 8,00 | 7,00 | 7,00 | 3,00 | 3,00 | 1,00 | 2,00 | 1,00 | 5,00 | 6,00 | 1,00 | 0,00 | 0,00 | 7,00 | 1,00 | 7,00 | 8,00 | 6,00 | 5,00 | 6,00 | 5,00 | 6,00 | 5,00 | 6,00 | 69,00 | 69,70 |
| C080 | Italy | E | 7,00 | 7,00 | 7,00 | 5,00 | 4,00 | 3,00 | 1,00 | 5,00 | 1,00 | 6,00 | 7,00 | 2,00 | 0,00 | 0,00 | 7,00 | 0,00 | 6,00 | 7,00 | 7,00 | 7,00 | 4,00 | 6,00 | 6,00 | 6,00 | 6,00 | 72,00 | 72,73 |
| C081 | Italy | E | 8,00 | 7,00 | 6,00 | 5,00 | 3,00 | 3,00 | 3,00 | 3,00 | 1,00 | 4,00 | 6,00 | 3,00 | 1,00 | 0,00 | 5,00 | 1,00 | 6,00 | 6,00 | 5,00 | 5,00 | 5,00 | 4,00 | 4,00 | 4,00 | 5,00 | 57,00 | 57,58 |
| C087 | Italy | E | 6,50 | 8,00 | 6,50 | 6,00 | 4,00 | 2,00 | 1,00 | 6,00 | 1,50 | 3,00 | 5,50 | 2,00 | 0,50 | 1,00 | 6,50 | 2,00 | 6,00 | 7,50 | 6,50 | 6,50 | 6,00 | 6,00 | 6,50 | 6,50 | 6,00 | 73,50 | 74,24 |
| C106 | Italy | E | 8,00 | 7,00 | 7,00 | 7,00 | 5,00 | 4,00 | 1,00 | 5,00 | 1,00 | 6,00 | 6,00 | 1,00 | 0,00 | 0,00 | 7,00 | 1,00 | 7,00 | 7,00 | 7,00 | 6,00 | 5,00 | 6,00 | 7,00 | 7,00 | 7,00 | 77,00 | 77,78 |
| C138 | Italy | E | 7,00 | 7,00 | 7,00 | 7,00 | 3,50 | 4,00 | 1,00 | 4,00 | 2,00 | 4,00 | 5,00 | 1,00 | 0,00 | 0,00 | 6,50 | 0,50 | 6,50 | 7,00 | 6,50 | 6,00 | 6,00 | 6,50 | 7,00 | 6,50 | 7,00 | 74,50 | 75,25 |
| C140 | Italy | A | 7,00 | 0,00 | 6,00 | 5,00 | 1,00 | 3,00 | 2,00 | 2,00 | 1,00 | 3,00 | 5,00 | 1,00 | 1,00 | 1,00 | 3,00 | 3,00 | 5,00 | 7,00 | 5,00 | 6,00 | 4,00 | 4,00 | 3,00 | 3,00 | 3,00 | 44,00 | 44,44 |
| C143 | Italy | E | 7,00 | 6,50 | 6,50 | 6,50 | 4,00 | 4,00 | 1,50 | 4,00 | 1,00 | 5,50 | 5,50 | 1,50 | 0,00 | 0,00 | 6,50 | 1,00 | 7,00 | 5,50 | 6,50 | 6,50 | 5,50 | 5,50 | 6,00 | 6,00 | 6,50 | 70,50 | 71,21 |
| C149 | Russian Federation | F | 6,00 | 7,00 | 6,00 | 4,00 | 4,00 | 4,00 | 2,00 | 2,00 | 2,00 | 5,00 | 4,00 | 1,00 | 0,00 | 0,00 | 7,00 | 1,00 | 6,00 | 6,00 | 6,00 | 4,00 | 6,00 | 5,00 | 5,00 | 5,00 | 6,00 | 61,00 | 61,62 |
| C154 | Slovenia | F | 8,00 | 7,50 | 7,50 | 7,00 | 5,50 | 3,50 | 2,00 | 4,00 | 2,00 | 4,50 | 5,50 | 2,00 | 0,00 | 0,00 | 7,00 | 1,00 | 7,00 | 7,50 | 6,50 | 7,00 | 5,00 | 6,00 | 6,00 | 6,00 | 6,00 | 73,00 | 73,74 |
| C161 | Spain | F | 6,50 | 7,50 | 6,00 | 4,50 | 6,00 | 2,00 | 0,00 | 6,00 | 0,50 | 4,00 | 3,50 | 2,00 | 0,00 | 0,00 | 7,50 | 0,50 | 7,00 | 6,00 | 6,50 | 6,00 | 3,50 | 4,50 | 6,50 | 5,50 | 7,00 | 67,50 | 68,18 |
| C162 | Spain | F | 7,00 | 6,00 | 7,00 | 6,00 | 6,00 | 2,00 | 0,00 | 6,00 | 1,00 | 3,00 | 4,00 | 1,00 | 0,00 | 0,00 | 8,00 | 1,00 | 8,00 | 6,00 | 8,00 | 8,00 | 6,00 | 7,00 | 8,00 | 7,00 | 7,00 | 78,00 | 78,79 |
| C163 | Spain | F | 6,00 | 6,50 | 6,50 | 6,00 | 5,50 | 4,00 | 1,50 | 5,00 | 1,50 | 3,50 | 3,50 | 1,00 | 0,00 | 0,00 | 7,00 | 1,00 | 6,00 | 6,00 | 7,00 | 4,50 | 5,50 | 5,50 | 6,00 | 6,50 | 5,50 | 67,50 | 68,18 |
| C167 | Spain | F | 7,00 | 7,00 | 8,00 | 6,00 | 6,00 | 2,00 | 1,00 | 6,00 | 0,00 | 3,00 | 4,00 | 2,00 | 0,00 | 0,00 | 6,00 | 1,00 | 6,00 | 7,00 | 7,00 | 7,00 | 5,00 | 5,00 | 6,00 | 6,00 | 6,00 | 71,00 | 71,72 |
| C168 | Spain | F | 5,00 | 7,00 | 8,00 | 6,00 | 6,00 | 3,00 | 1,00 | 7,00 | 2,00 | 3,00 | 3,00 | 2,00 | 0,00 | 0,00 | 8,00 | 1,00 | 7,00 | 5,00 | 8,00 | 7,00 | 5,00 | 6,00 | 7,00 | 8,00 | 6,00 | 75,00 | 75,76 |
| C169 | Spain | F | 6,00 | 6,00 | 7,00 | 4,00 | 5,00 | 2,00 | 1,00 | 3,00 | 1,00 | 5,00 | 6,00 | 2,00 | 0,00 | 1,00 | 6,00 | 1,00 | 7,00 | 5,00 | 5,00 | 6,00 | 5,00 | 4,00 | 6,00 | 5,00 | 6,00 | 59,00 | 59,60 |
| C171 | Svizzera | F | 7,00 | 7,00 | 7,00 | 6,00 | 5,00 | 3,00 | 1,00 | 6,00 | 0,00 | 4,00 | 6,00 | 2,00 | 0,00 | 0,00 | 7,00 | 1,00 | 7,00 | 8,00 | 6,00 | 6,00 | 7,00 | 7,00 | 7,00 | 6,00 | 7,00 | 77,00 | 77,78 |
| C183 | Perù | B | 7,00 | 0,00 | 8,00 | 4,00 | 4,00 | 1,00 | 1,00 | 5,00 | 1,00 | 4,00 | 4,00 | 1,00 | 0,00 | 0,00 | 7,00 | 0,00 | 6,00 | 6,00 | 8,00 | 7,00 | 6,00 | 6,00 | 8,00 | 7,00 | 8,00 | 69,00 | 69,70 |
| C199 | Japan | A | 7,00 | 0,00 | 7,00 | 4,00 | 1,00 | 2,00 | 0,00 | 4,00 | 1,00 | 3,00 | 2,00 | 1,00 | 0,00 | 0,00 | 4,00 | 0,00 | 6,00 | 8,00 | 6,00 | 7,00 | 5,00 | 4,00 | 3,00 | 4,00 | 5,00 | 53,00 | 53,54 |
| C204 | Taiwan | F | 5,50 | 8,00 | 6,50 | 5,00 | 6,50 | 2,50 | 2,00 | 7,00 | 2,50 | 2,50 | 4,00 | 1,00 | 0,00 | 2,00 | 6,00 | 1,50 | 6,00 | 5,50 | 5,50 | 6,00 | 4,50 | 3,50 | 6,50 | 5,50 | 6,00 | 63,50 | 64,14 |
| C208 | Taiwan | A | 9,00 | 0,00 | 6,00 | 2,00 | 4,00 | 1,00 | 0,00 | 4,00 | 1,00 | 4,00 | 4,00 | 1,00 | 0,00 | 0,00 | 6,00 | 0,00 | 6,00 | 5,00 | 6,00 | 6,00 | 6,00 | 4,00 | 6,00 | 5,00 | 6,00 | 54,00 | 54,55 |
| C222 | Taiwan | F | 7,00 | 8,00 | 7,00 | 5,00 | 7,00 | 2,00 | 1,00 | 7,00 | 1,00 | 3,00 | 3,00 | 2,00 | 0,00 | 1,00 | 6,00 | 1,00 | 6,00 | 5,00 | 6,00 | 5,00 | 4,00 | 5,00 | 6,00 | 6,00 | 5,00 | 64,00 | 64,65 |
| C225 | Taiwan | F | 7,00 | 8,00 | 7,00 | 6,50 | 3,50 | 4,00 | 1,50 | 5,50 | 2,00 | 4,00 | 5,50 | 2,00 | 0,50 | 2,00 | 6,50 | 2,00 | 6,50 | 7,50 | 6,50 | 6,00 | 5,50 | 5,50 | 5,50 | 6,00 | 6,00 | 70,50 | 71,21 |
| C236 | Taiwan | F | 7,00 | 7,00 | 7,00 | 6,00 | 4,00 | 3,00 | 2,00 | 3,00 | 2,00 | 4,00 | 6,00 | 1,00 | 0,00 | 1,00 | 6,00 | 2,00 | 6,00 | 7,00 | 7,00 | 6,00 | 6,00 | 5,00 | 7,00 | 6,00 | 6,00 | 69,00 | 69,70 |
| C250 | China | F | 8,00 | 7,50 | 7,50 | 7,00 | 4,00 | 3,50 | 1,00 | 3,00 | 2,00 | 4,00 | 6,50 | 2,00 | 0,50 | 0,50 | 7,00 | 2,50 | 7,00 | 8,00 | 7,50 | 7,50 | 5,50 | 6,50 | 6,00 | 6,00 | 6,00 | 74,00 | 74,75 |
| C287 | China | A | 6,00 | 0,00 | 5,00 | 2,00 | 4,00 | 2,00 | 0,00 | 6,00 | 0,00 | 2,00 | 5,00 | 2,00 | 0,00 | 0,00 | 7,00 | 1,00 | 5,00 | 4,00 | 5,00 | 7,00 | 6,00 | 3,00 | 7,00 | 7,00 | 7,00 | 56,00 | 56,57 |
| C297 | China | F | 7,00 | 7,00 | 7,00 | 4,00 | 4,00 | 4,00 | 1,00 | 3,00 | 2,00 | 4,00 | 5,00 | 1,00 | 0,00 | 1,00 | 7,00 | 1,00 | 7,00 | 7,00 | 7,00 | 6,00 | 6,00 | 6,00 | 6,00 | 6,00 | 6,00 | 67,00 | 67,68 |
| C304 | Korea | A | 7,00 | 0,00 | 5,00 | 4,00 | 4,00 | 1,00 | 0,00 | 6,00 | 1,00 | 3,00 | 4,00 | 1,00 | 0,00 | 0,00 | 7,00 | 0,00 | 7,00 | 6,00 | 7,00 | 7,00 | 6,00 | 6,00 | 8,00 | 7,00 | 7,00 | 67,00 | 67,68 |
| C313 | Korea | F | 7,50 | 7,00 | 7,00 | 7,00 | 4,00 | 5,50 | 1,00 | 4,00 | 1,00 | 4,50 | 4,50 | 1,50 | 0,00 | 0,00 | 7,00 | 1,00 | 7,00 | 7,00 | 7,00 | 6,00 | 6,00 | 6,00 | 6,50 | 6,50 | 6,50 | 74,00 | 74,75 |
| C321 | Thailand | A | 5,00 | 0,00 | 5,00 | 1,00 | 3,00 | 1,00 | 0,00 | 4,00 | 2,00 | 1,00 | 1,00 | 1,00 | 0,00 | 1,00 | 5,00 | 1,00 | 2,00 | 5,00 | 5,00 | 5,00 | 6,00 | 5,00 | 4,00 | 4,00 | 4,00 | 43,00 | 43,43 |
