# Supplementary material for: Identification of markers of sensory quality in ground coffee: an untargeted metabolomics approach
Source: Metabolomics. 2020 Dec 14;16(12):127. doi: 10.1007/s11306-020-01751-6 (PMC7736008; doi:10.1007/s11306-020-01751-6)
Supplement: Supplementary file 7 — Supplementary file7 (DOCX 15 kb) [file 11306_2020_1751_MOESM7_ESM.docx]

**SUPPLEMENTARY MATERIAL**

**Supplementary table 1.** Details of the sensory tests performed at the International Coffee Testing 2018, including the sensory attributes investigated and the individual score of the different samples.

**Supplementary table 2.** UHPLC-QTOF-MS datasets containing the extracted molecular features (MFE), compounds identified by both MS-only using different databases (i.e. FoodDB, Phenol-Explorer, and Maillard reaction product) and MSMS (using MS-DIAL and MS-FINDER softwares on Quality Control samples) approaches and volatile compounds identified by HS-GC/MS. A principal component analysis (PCA) showing the overall variability of quality controls across sample groups is also provided.

**Supplementary table 3.** Semi-quantitative results on phenolic compounds and OPLS-DA model built considering the annotation from Phenol-Explorer database.

**Supplementary table 4**. Pearson's correlation coefficients considering VIP marker compounds (Table 1 and Table 2) and sensory descriptors.

**Supplementary figure 1.** Unsupervised hierarchical cluster analysis (HCA) heat map built considering the molecular features (MFs) annotated by UHPLC-QTOF-MS. (similarity: Euclidean; linkage rule: Ward). MFs' intensity was used to build up heat map, on the basis of which the clusters were generated.

**Supplementary figure 2.** Orthogonal Projections to Latent Structures Discriminant Analysis (OPLS-DA) score plot for high *vs* low quality coffee samples. The output was built considering the molecular features annotated by UHPLC-QTOF-MS. Besides, the R^2^Y and Q^2^Y predictive parameters are also reported.
